# Housing and Health of Kiribati Migrants Living in New Zealand

**DOI:** 10.3390/ijerph14101237

**Published:** 2017-10-17

**Authors:** Mary Anne Teariki

**Affiliations:** He Kainga Oranga/Housing and Health Research Programme, Department of Public Health, University of Otago, Wellington 6242, New Zealand; maryanne.teariki@otago.ac.nz

**Keywords:** housing, settlement, Kiribati migrants, health and well-being

## Abstract

Settlement is a complex process of adjustment for migrants and refugees. Drawing on recent research on the settlement experiences of Kiribati migrants and their families living in New Zealand, this article examines the role of housing as an influencer of the settlement and health of Kiribati migrants. Using qualitative methodology, in-depth interviews were conducted with fourteen Kiribati migrants (eight women and six men) representing 91 family members about the key issues and events that shaped their settlement in New Zealand. The stories told by participants affirm the association between housing and health. The study serves as an important reminder that children bear a great cost from living in poorly insulated and damp housing, and adults bear the mental costs, including social isolation resulting from inadequate rental housing. Detailed information about how this migrant group entered the private rental housing market, by taking over the rental leases of other Kiribati migrants vacating their rental properties, indicated some of the unintended consequences related to a lack of incentives for landlords to make improvements. With the most vulnerable families most at risk from inadequate housing, this research concludes that there is a need for minimum housing standards to protect tenants.

## 1. Introduction

With a history of strong constitutional and political relationships with the nations of the Pacific, New Zealand has always been an attractive migration destination for Pacific citizens. Despite a rich literature on the settlement and health of Pacific migrants living in New Zealand [1], little was known on settlement experiences of Kiribati migrants and their families, a relatively new migrant group to New Zealand. While remedying this knowledge gap was a significant impetus for this research, the likelihood of increased outward migration from Kiribati to New Zealand due to climate change and other anthropogenic impacts added an important impetus to this study [2].

Previous research on the key issues and events that shaped the settlement of Kiribati migrants living in New Zealand shows that housing in an important influencer of the settlement experiences of Kiribati migrants and their families [3]. It determines where families live, how they live, where their children go to school, and their physical and mental health. This article examines how Kiribati migrants moved into the New Zealand private rental housing market, and the unintended consequences of taking over the rental leases of other Kiribati families vacating their rental properties. It also explores the relationship between inadequate housing and health, including the role of language proficiency and the development of relationships between renters and landlords [3]. To ensure that the stories told by participants are heard, this article uses direct quotes to give meaning to the lived experiences of Kiribati migrants. Finally, this research discusses the merits of implementing minimum housing standards in the private rental housing market to protect renters.

This article is framed into four key parts. The first section investigates the demographic profile of the Kiribati population in New Zealand, and the contribution of immigration on the growth of the Kiribati population. The second section sets out the criteria used to identify the purposeful sample, and the method used to gather the rich data from participants. This section also discusses the author’s ‘insider/outsider’ status as a member of the Kiribati community for the past thirty years. The third section discusses the key findings of this research, focusing on the impact of inadequate housing on the health of children and adults. The fourth section compares these findings with other research, focusing on how best to protect the most vulnerable families from inadequate housing.

## 2. Kiribati Population of New Zealand

New Zealand has long been an important migration destination for citizens from the Pacific. Historically, the migration of Pacific peoples to New Zealand has been influenced by a range of constitutional and political relationships, and the demand for labour in the post-World War II period. Nowadays, inward migration from this region has been significantly influenced by specific immigration policies geared towards the Pacific. For Kiribati citizens, the Pacific Access Category (PAC) that was implemented in 2003 has provided a key migration route to permanently live in New Zealand. Through this immigration policy, up to 75 citizens per year, successfully balloted and meeting employment, income, health, and character requirements may be invited to apply for residence [4]. With the PAC providing a key immigration route for I-Kiribati into New Zealand, a younger demographic profile, and inclination for larger families, the Kiribati ethnic population grew by 89.5 percent from 2006 to 2013 [5].

An examination of the 2013 Census identified that the Kiribati ethnic group was comprised of 2115 people living in New Zealand [5]. Of this group, 93 percent lived in the North Island, with 75.6 percent of those living in the main urban centres (populations of 30,000 or more). The most common regions to live were the Auckland region (45 percent), followed by the Waikato region (17.9 percent) and the Wellington region (13.9 percent). The 2013 Census also confirmed that this ethnic population is largely a migrant group with 67.2 percent being born overseas and 32.8 percent born in New Zealand. Of those born overseas, 36.3 percent had arrived in New Zealand less than five years ago, compared with 52.8 percent in 2006. Consistent with the profile of the Pacific ethnic group living in New Zealand, the Kiribati ethnic group is significantly younger than the New Zealand population, with 37.9 percent of the Kiribati ethnic group under the age of 15 years in 2013, compared to 20.4 percent for the total New Zealand population.

The 2013 Census also shows that the Kiribati ethnic group is concentrated in low-paying industries, working as labourers (35 percent), and community and personal service workers, such as, caregivers (18.1 percent). Consistent with this employment profile, 56 percent of the Kiribati ethnic population aged 15 years and older received an annual income of less than $20,000, with only 3.6 percent receiving an annual income of more than $70,000. This low profile is also depicted in the median income (half received less and half received more income) identified at $14,700 in 2013, a reduction from $18,000 in 2006. In line with these income indicators, only 11 percent of the Kiribati populations are identified as owning, or partly owning their homes in 2013, compared to 18.5 percent for Pacific peoples, and 49.8 percent for the New Zealand population. Of those renting, 80.8 percent were identified as living in dwellings with a private landlord, compared to 56.3 percent for Pacific peoples [5]. This difference can be explained by the significantly lower take-up of state-assisted rental housing between I-Kiribati and the Pacific population as a whole. With 80.8 percent of the Kiribati ethnic group living in the private rental sector, only 11.6 percent of I-Kiribati were identified as living in state-assisted housing, compared to 41 percent of the Pacific population (led by Samoan, Tongan, Tokelauan, and Cook Islander ethnic groups) living in state housing. In recognition of the increasing problem of affordability facing many New Zealand families, particularly Māori and Pacific households, Bierre et al. (2013) have argued that there is a need for policies and programmes to increase the proportion of social and affordable housing, as a proportion of the total New Zealand housing stock [6].

The demographic profile of the Kiribati ethnic group in New Zealand suggests that I-Kiribati are likely to face a number of risks associated to affordability and their high level of concentration in the largely unregulated private rental housing sector. With a lack of minimum health standards in the private rental market, evidence on the detrimental impact of inadequate housing on the health of Pacific families [7], suggests that Kiribati families living in poorly heated and poorly ventilated rental houses, are likely to faced increased health risks, particularly in children exposed to several allergens commonly found in homes, such as, moulds and dust mites. These factors, coupled with a higher proportion of Pacific children and young people living in over-crowded households, have been linked with higher levels of respiratory diseases, asthma, skin infections, tuberculosis, and meningococcal disease. Consistent with these findings, research from New Zealand and the United States note the positive health impacts from the retrofitting of insulation in existing homes. In New Zealand, retrofitted insulation was associated with lower self-reported: days off school and work, visits to doctors, and hospital admissions for respiratory diseases [8]. In the United States, housing improvements, and the use of multi-component approaches (rather than single interventions) were associated with improved health outcomes [9]. Adding to these complexities, cumulative negative health outcomes have been identified for migrants, if inadequate housing is found to exist with low language proficiency and limited social networks [10]. It is within this rich body of research that this article examines the relationship between housing and the health of Kiribati migrant families.

## 3. Materials and Methods

### 3.1. Constructivist Grounded Theory

This research uses qualitative methodology to gather and analyse data collected from participants on their settlement and housing experiences in New Zealand. Using constructivist grounded theory, the aim was to understand the meaning of peoples’ lived experiences, not only to understand the actions undertaken by participants and their families during their settlement, but to try and comprehend why they took those actions, and what those experiences meant to them [11]. To be in a position to gather collect this level of detail, considerable focus was placed on identifying the sampling frame and inclusions, the best way to inform members of the Kiribati community of the research, and how to frame the interviews to make participants feel comfortable in sharing their settlement stories.

### 3.2. Sampling Frame and Inclusions

To analyse the settlement experiences of Kiribati migrants and their families living in New Zealand, the sampling frame was identified as those within the Kiribati ethnic population of New Zealand who had migrated to live in New Zealand, rather than those born in New Zealand. Of the 1401 I-Kiribati identified as having been born overseas in the 2013 New Zealand Census [6], the 86.9 percent identified as having been born in Kiribati, (1217 people), were those identified as comprising the sampling frame. A number of inclusions were considered necessary to identify the sample for this research. With the emphasis of the research on the settlement experiences of Kiribati migrants, a key inclusion was for the sample to comprise of Kiribati people who entered New Zealand on a Kiribati passport, and who had migrated from Kiribati, rather than a third country. The reason for this was to understand the adjustment of those migrating from Kiribati to New Zealand. In addition, with the focus on settlement, another important inclusion was for those in the sample to have gained permanent residence, rather than holding a temporary visa.

Another important consideration was to include those Kiribati migrants with permanent residence who had lived in New Zealand less than ten years. While this could have been set closer to five years (to capture a sense of newness to New Zealand), the evidence from the literature that settlement could involve a much longer process (even generational or life-long for some migrants), pointed to the wisdom of setting a longer settlement timeline of less than ten years [12]. With the focus on the settlement experiences of Kiribati migrants and their families, it was considered appropriate to have the sample comprise of Kiribati adults (aged 18 years and over) with dependent children. The inclusion of children was to ensure that this research captured the fuller depiction of complexities associated with the settlement of Kiribati families. Finally, with the focus on conducting in-depth face-to-face interviews, the restriction to the wider-Wellington geographical area for this study, facilitated giving feedback to participants in person.

In summary, the purposeful sample comprised of those who were:

- Adults, aged 18 years or older, with one of more dependent children; and who
- Migrated to New Zealand as Kiribati citizens; and who
- Migrated to New Zealand from Kiribati; and who
- Where granted residence status to live in New Zealand; and who
- Lived in New Zealand for less than ten years (including any time spent in New Zealand prior to gaining residence); and who
- Resided in the wider-Wellington region.

### 3.3. Connecting with the Wellington Kiribati Community (WKC)

As a member of the WKC, the formal body representing Kiribati families living in the wider-Wellington region, the leader of this organization was approached to assist me to communicate the intent and process for this research. A written information sheet, written in Kiribati and English was circulated by the President of the WKC to its members, inviting those meeting the criteria to participate. Through a process of self-selection and cold-calling, 14 participants (eight women and six men), representing 91 family members, living in the wider-Wellington region were identified as the participant group. The small size of the group provided an opportunity to dig deeper on issues of interest, including exploring in detail how participants felt about those experiences. While generalizing from the settlement experiences of Kiribati migrants living in an urban area does not necessarily mirror the settlement experiences of Kiribati migrants living in larger cities, or those living in more rural areas, the insights gained in this qualitative study are informative.

### 3.4. Interview Method

Taking account of social and cultural norms, the conversational interview method was used to gather information from participants about their housing experiences during their settlement in New Zealand. To enable participants to tell their own stories, the interviews were structured along the lines of each participant’s actual settlement journey. This allowed for rich data to be gathered on the key events that shaped the settlement experiences of each participant, starting with their decision to migrate to New Zealand, their expectations, their first experiences upon landing at Auckland International Airport, their first and subsequent housing experiences, to where they find themselves today. This structure enabled me to dig further on particular issues, such as, their living environment and how those environmental factors may have influenced the health of their family. With housing recognised as a social determinant of health, participants were asked to describe the state of their homes, and what they observed regarding the health of their family, particularly their children.

Set against epidemiological evidence of indoor environmental factors influencing health outcomes [13], including the relationship between cold living environmental, dampness, moulds and other allergens, participants were questioned about their health experiences and patterns of illness during the winter months. Based on their stories, diagnoses from primary health professionals, hospitalisations, and how the health of their families changed when they changed their living environments, links were observed between participants’ health stories and the state of their home environments. Despite difficulties in identifying all the factors influencing the health of participants and their families, the observations made by participants were consistent with the large body of evidence linking inadequate housing with detrimental health outcomes.

### 3.5. Insider/Outsider Status

An important aspect of the methodology, used to gather data from Kiribati migrants, was my insider/outsider status with the WKC. Being married to my I-Kiribati husband for 30 years, and a member of the WKC, I was connected to the Kiribati community within which we circulated in. Having lived in Kiribati and having accumulated knowledge of the cultural and social customs of the Kiribati way of life, I had a good understanding of the stories being told and how they were being conveyed. My understanding and empathy for the Kiribati culture and its people led participants to feel close to me, with participants noting that I knew ‘the Kiribati way’, and that I was I-Kiribati. While not seeking to take this for granted, these experiences led me to believe that I held some of the advantages of being an insider. One of the most significant advantages of these close connections was my ability to understand what people were trying to convey to me, particularly when the replies to my questions were broken into English and Kiribati.

Alongside the many benefits garnered by being treated as an ‘insider’, perversely being also an ‘outsider’ may have also influenced the level of openness displayed during the interviews. The rationale for this conjecture is that innermost reflections particularly relating to difficulties are almost never shared outside the realm of the family group. Being an ‘outsider’ is likely to have assured participants that there was no purpose in gathering information on their settlement experiences other than for this research. Reflecting on this experience, I considered that being both a ‘insider’ and ‘outsider’ assisted participants to share a wider level of information, compared to if I was either just an ‘insider’ or an ‘outsider. Being an ‘outsider’ with a great love of Kiribati, its people and culture, is likely to have come together to generate the greatest influence in the willingness of participants to share their settlement experiences.

## 4. Results

Consistent with the interview approach, this section examines the key issues raised by participants regarding their housing experiences in New Zealand. The structure of the section depicts the lived experiences of participants and their families, starting with their earliest experiences of being accommodated in the homes of family and friends, followed by their first and subsequent rental housing experiences and their impact on health, and finishing with the transition for two participants from rental housing to home ownership.

### 4.1. Early Housing Experiences

The earliest housing experiences recalled by most participants, was living with family and friends when they first arrived in New Zealand. With no job and income, and uncertainty of immigration status, participants told of their deep appreciation of being taken in by their social networks. With unrealistic expectations of the type of housing they would experience, many participants (led by women) spoke of their shock and surprise at the quality of rental housing in New Zealand. Much of this shock and surprise was attributed to the small size and closed nature of homes, and the significant level of over-crowding being experienced by Kiribati migrants. Unlike the open homes of Kiribati that enabled extended families come and to live together [14], the typical New Zealand home of three bedrooms and one bathroom was viewed as being inadequate for large Kiribati families, let alone when assisting new Kiribati migrants settle in New Zealand. This was depicted by one female participant who spoke of her shock upon realising that she and her daughter would be sharing a bedroom with eight other people:

*“When we first arrive and the family … pick us there and when we came there we thought that’s a big house … but when we get there we just realise that it’s one bedroom … ten people one bedroom … they nice family.” (# younger aged female*, *married*, *small family*, *8 years settlement)*

This direct quote reflects a number of sentiments commonly expressed by participants, ranging from shock and surprise at the living conditions of many Kiribati families living in New Zealand to deep gratitude at the willingness of these families to take them into their homes. Many participants, in particular, women acknowledged the efforts made by their social networks to accommodate, feed them, and assist them during the early stages of their settlement. At the same time, these participants expressed concerns about the effects of inadequate housing and over-crowded conditions on the health of their children. Among the most common health issues identified by participants were a range of respiratory illness, including asthma, and skin infections requiring medical intervention. Participants recalled the speed with which household members became ill, particularly during the winter months, when family members congregated around one heating appliance in the living room. While living in an extended family situation reminded many participants of the communal style of living back in Kiribati, the small and closed structure of New Zealand houses made for difficult living arrangements. Over time, these problems became a source of significant stress for some participants, particularly women, who felt that their families were becoming a burden on their relatives, but who had few options but to continue living with them until they gained full-time, permanent employment to establish their own households. Amongst these difficulties, some women highlighted a number of benefits of living with their familial networks, particularly in terms of observing the daily routines of family life in New Zealand. This was described by one female participant:

*“… I just go with the flow with their routine … both working at that time you know so the kids you know went to school and they both work together … I observe what is happening every day … they were like you know, in the morning they have to get up early, prepare their kids to school get their lunch …” (# middle aged female*, *married*, *medium sized family*, *7 years settlement)*

### 4.2. Entry into the Private Rental Market

Contrary to a large body of literature on the housing of migrants and refugees [15], entry into their first rental properties was described by all participants as being a relatively straightforward process. This can be attributed to the widespread assistance provided to participants from family, friends, and other social networks, such as, church contacts. Digging further in this issue it also became apparent that the unconventional manner that participants used to secure their first rental properties played a major part in the ease with which they moved into rental properties. With limited incomes, and armed with little knowledge on how the private rental market worked in New Zealand, half of the participant group relied on their social networks to get them into the rental housing market. Rather than going through the usual process of applying for rental properties, these social networks obtained information when other Kiribati families were about to vacate their rental properties, and advised participants to contact these families so that they could get ahead of the selection process. This process enabled seven families to take over the rental leases of other Kiribati families vacating their rental properties, without the property ever going to the market. This process enabled participants, who had little or no knowledge of the rental housing market, and who were unable to provide written references on their rental housing experience to secure their first rental homes. One participant recalled how this process worked for her family:

*“I have to ask for … a friend for a house to help me … it is a Kiribati friend too and you know that Kiribati lived in the house before and they’re going to move out from that house. So I just went to that lady and ask her “would you please ask your landlord if I can stay there?” Yes so it’s really hard for me to hunt for a house, I think that is the best way that I can get a house just to go and ask her cos they moving out and so just to step in that house.” (# middle aged female*, *married*, *medium sized family*, *7 years settlement)*

Heralded as one of the most significant events of their settlement in New Zealand, this process was hailed by these participants as helping them reach their goal of establishing their own independent households. Unsurprisingly, this process of ‘flipping leases’ was also favoured by rental property owners, given that it eliminated the costs of having to advertise their properties, and significantly reduced the period that these properties would need to lay vacant. While at the time, this process was viewed by participants as being a ‘win-win’ situation for them and property owners, the consequences of this process became apparent a short time after participants moved into their rental homes. By eliminating the need for owners to make improvements to their rental properties to have them rented, these families came to realise the social and detrimental health costs of living in inadequate housing. These seven families lamented the poor state of their rental properties, typically described as being very cold and damp, and the impacts of this environment on the health of their children. Participants told of their children becoming ill, particularly during the winter months, with a range of respiratory illnesses, including asthma and chest infections. Some participants also noted their children suffered from recurring skin infections requiring medical intervention.

Notwithstanding the poor state of their rental properties, many participants recalled the benefit of living in close proximity to family and friends, during that phase of their settlement. With limited resources, and with both adults in the family working, one participant depicted how wider family connections assisted him with respect to his children:

*“… was just a few blocks away from my auntie’s so every now and then she could come and help with the kid as well especially cos we only got one vehicle at that time so … sometimes I work and there’s no one to pick up my kids so they have to walk and if it’s raining then eh, then I have to call my aunty to pick them up you know they just attend the school … I want to stay close to my aunty so, you know, so she could help out with transportation and also with my immigration papers” (# younger aged male*, *married*, *medium sized family*, *six years settlement)*

Aside from the benefits of living near relatives and friends, most participants recalled their expectations of being able to move on from their first rental properties after a short period, and move into better quality rental housing. While this was possible for some families, the next section discusses the implications of inadequate housing on some of the most vulnerable participants and their families.

### 4.3. Quality of Housing

While most participants described their first rental properties as being inadequate, most expected that they would be able to move into better rental housing as they moved into higher paying jobs. This was not, however, the case for those families where both adults worked in low-wage employment, with little prospect of upward labour market mobility. Expectedly, these income disparities resulted in significantly different housing experiences across the participant group. For example, in contrast to those participants who moved into higher paying jobs (as marine engineers, seafarers, nurses, and public servant) and who could afford to move into better quality rental properties, or in two cases, purchases their own homes, those families with low-incomes had few choices of where they could live. With limited financial resources, three participants felt that they had few options but to continue to live in the rental houses that had been vacated by other Kiribati families. Although these houses were identified by participants as being inadequate, their relationships with their landlords materialised as a key influencer of whether their housing experiences would improve.

In one example, a female participant described her success in convincing her landlord to improve the heating of her rental property, arguing that the lack of heating was having an adverse impact on the health of her children. By way of contrast, a male participant described how his requests to get his landlord to improve his cold, damp, and pest infested house were repeatedly declined. In this case, the landlord went further to threaten the participant, noting that if was to vacate his rental property, he would be expected to pay for the wear and tear of the property. By effectively imposing exit costs, this landlord took advantage of someone who could not speak English, and who did not know his rights as a tenant. On this occasion, the participant worried greatly about the health of his children and attributed his poor mental health on his inability to provide his family with adequate housing. The divergent experiences of these two participants:

*“Like I don’t care about the cost more but I have to focus on my health and my family. He working now on the windows, working now on the insulating the house … apart from that you know toilets and showers, kitchen is the most important parts so he has to focus on that … that landlord is good. My older daughter is having asthma before but now like I couldn’t hear her coughing a lot now which helps a lot …” (# middle aged female*, *married*, *medium sized family*, *7 years settlement)*

*“... we found that all the cockroaches … we talk to the landlord but the landlord … says … that’s not my job to do that, you … must do that one because you bring the cockroach. We want to bring our friends but we shy with the house … it’s very old and I said oh no you can bring them but just eat and they leave like that.” (# middle aged male*, *married*, *very large family*, *7 years settlement)*

While the personal attitudes of landlords can be credited for the divergent housing experiences of participants, this research raises the possibility that language proficiency may also have played a part in the ability of participants and landlords to build effective relationships. Although the role of language proficiency is unclear, the manner in which the last participant was treated and threatened with exit costs and legal proceedings, strongly suggests that his landlord took advantage of this participant’s poor English language skills and, consequently, his ability to contest the assertions made by his landlord. The experience of this participant, and the living conditions of those participants, who took over the leases of other Kiribati families, suggests that current regulatory frameworks are inadequate in enforcing minimum standards in the private rental housing sector. While robust discussion has begun in New Zealand on the pros and cons of regulations enforcing a minimum standard (with one local government council deciding to implement a Warrant of Fitness for rental properties), the lack of political conviction at the government level, has led to the continuing renting of inadequate rental properties to the most at risk families.

Adding to this complexity, is the need to ensure that the implementation of any new standards for rental housing covers those rental properties swapped from one family to another, as was the case with half of the participant group. This churn at the bottom of the private rental market poses a significant challenge, given the propensity, as indicated in this research, for prospective renters to take on any rental property, in the hope that improvements might be made at a later date, or that their time in these properties are temporary. An interesting finding of this research was fact that none of the most vulnerable families had attempted to seek social housing assistance. Probing further on the issue, it became apparent that these families did not know how to go about asking for assistance, and lacked the courage to approach social housing institutions. Once again, poor language proficiency emerged as a barrier to these families in seeking out information about social services available to them. Although this is likely to improve as more Kiribati migrants gain knowledge of the processes they need to undertake to be considered for social housing, more could be done to reach out to these vulnerable families, including the provision of information written in the Kiribati language.

### 4.4. Home Ownership

Home ownership was heralded by all participants as one of the most important long-term aspirations of their settlement, with many participants indicating their desire to start a savings plan to enable them to put a deposit on a house. Despite this, only two participants of the group of fourteen had succeeded in making the transition from rental properties to purchasing their own homes. In one case, a male participant and his partner, both of whom worked in career employment, told of how they had been able to save for their deposit to purchase their home. With a small family, and a good combined income, this participant recalled how he and his wife were able to secure a mortgage to finance the purchase of their home.

*“My wife and I decided to mortgage a house and we’re slowly paying it off, we earning wages all the time and keeping the family together you know.” (# middle aged male*, *married*, *small family*, *7 years settlement)*

In another case, a male participant told of the financial support he had received from his parents to raise a deposit to fund the purchase of their property. In this case, the participant noted that his home ownership goal would not have come to fruition had his parents not been in a financial position to assist his family. Working in a career job, this participant also recalled being successful in securing a mortgage to purchase his home.

*“Yeah, at first like when I purchased my house it was my parents you know that backed me up with all these like a deposit and everything even though I paid just part of it but most of it my parents so I think they really have a big impact on … helping us settle down in New Zealand yeah” (# younger aged male*, *married*, *medium sized family*, *six years settlement)*

In both of these cases, and right across the participant group, home ownership was considered as a key factor in participants and their families feeling a greater sense of attachment to their new host country. Other benefits of home ownership articulated by participants, included: keeping their families together, and allowing them to pass something tangible to pass to their children when they got old, and eventually passed away. These aspirations were articulated by two participants:

*“You feel like you belong yeah it’s the sense of belonging … right now I feel like I belong to New Zealand … cos I got my own house …” (# younger aged male*, *married*, *medium sized family*, *six years settlement)*

*“… we want to build our own house or buy our own house … cos we got a kid and yeah when we gone or pass away she can stay and not struggle with their future.” (# younger aged female*, *married*, *small family*, *8 years settlement)*

Given the value placed on owning their homes, it was unsurprising that the two participants, who had purchased their homes, expressed high levels of happiness about their settlement in New Zealand. This was despite one participant noting the burden of financing a mortgage was a source of stress and anxiety, linked to concerns about employment certainty, and the inability to take time off work to pursue further education that would have resulted in higher paying employment. Despite these concerns, both of these participants, and many others in the group saw home ownership as the pinnacle of settlement achievement. The downside of these strong sentiments is the possibility that those unable to afford to buy their own homes may view their settlement as a failure.

## 5. Discussion

In this section, the results from the data gathered is analysed against the emerging theory (based on evidenced-based research) that inadequate housing is associated with negative health outcomes [16]. In particular, the focus is on observations made by participants linking inadequate dwelling conditions and direct health effects, such as, the lack of heating systems and insulation with winter illnesses (respiratory infections and asthma). An important starting point for this discussion is that housing is a significant determiner of how Kiribati migrants go about their settlement. Housing determines, among other things, where families reside, how they live together, where children go to school, and proximity to their social networks. While it is recognised that a multitude of factors influence health outcomes, and that housing is one among a number of social determinants of health [17], the results of this research are consistent with the evidence of other studies that poor health outcomes is linked to inadequate housing. In particular, those participants who spoke of the poor health of their children associated high incidences of winter illnesses with the cold and damp conditions of their homes. These participants recalled how during the winter months, they and their families struggled to keep warm and how they congregated in one room, around a heater, or wrapped in blankets in an effort to keep warm.

The link between this pattern of over-crowding and the spread of respiratory, and other infectious diseases (such as, skin diseases, whooping cough, and tuberculosis) were also evident in this study. Hospitalisation from respiratory diseases and the rise of asthma attacks among children during winter, and days of school and work, were in line with research undertaken in New Zealand on the relationship between housing and health [16]. Similarly, the recollection by two male participants of the effects of inadequate housing and their mental health, underpinned the negative impacts that these participants associated with a lack of control over their lives, particularly in terms of their inability to provide their family with good housing [3]. Their inability to gain control and achieve the outcomes they desired was linked to their low self-esteem and their low sense of ‘belonging’ to New Zealand. While these, and other participants, were optimistic about the future, the inability to control the state of their living conditions and the health of their children weighed heavily on their view of failing as parents.

Finally, in terms of the where participants chose to live, the decision of most participants to move away from their social networks, but to stay within the general geographical location they had come to know, underpinned the notion of ‘familiarity’ as a key influencing factor of where participants and their families resided. The tendency of participants and their families to remain within the spatial location they had become familiar, even upon earning higher incomes, supports the findings of an inverse association between upward economic mobility and the tendency of Pacific peoples to live in Pacific dominated neighbourhoods [18]. While it could be argued that this inverse relationship amounts to segregation, or at the least, not supporting integration, these somewhat extreme explanations are less than convincing, given that the preferences of participants was about the sorts of neighbourhoods they enjoyed, rather than being a means to not connecting with other New Zealanders.

## 6. Conclusions

Settlement is a complex process of adjustment, starting from the time new migrants enter their new host country. While it is generally accepted that this process is influenced by a multiplicity of factors, this research highlights the importance of housing in providing Kiribati migrants with a sense of independence, stability, and aspiration for the future. Analysis of the stories told by participants, about their housing experience, indicated an association between inadequate housing and detrimental health effects. Most significantly, the description by participants of their families, particularly their children, suffering from respiratory illnesses and asthma, during the winter months was linked to the cold and damp conditions of their homes, resulting from a lack of adequate heating and ventilation. In particular, the stories depicting families living and sleeping in one room in an effort to keep warm during winter show-cased the poor living environment that many Kiribati families experienced during the early stages of their settlement. Conversely, the stories describing how their children’s health improved when they moved into warmer and drier homes, or when their landlords agreed to install heating and ventilation systems, also supported the likelihood of a link between housing and health. This research acknowledges that the study of housing and health is not an exact science, given that many factors influence the health of adults and children. At the same time, listening to the voices of Kiribati migrants living in New Zealand served as an important reminder that the quality of housing matters, most particularly, for vulnerable children. Even with all the caveats, this research serves an important reminder that children bear a great cost from inadequate housing, while adults bear the cost of living with mental anguish about their families living in poor quality housing. While one answer to this problem is to have these families move into social assisted housing, a better option would be to implement minimum standards for rental properties as a way of providing credible safeguards to renting in the private rental market.
